# CHARTING NEW PATHS: LGBTQIA+ GERIATRICS FELLOWSHIPS

**DOI:** 10.1093/geroni/igae098.3827

**Published:** 2024-12-31

**Authors:** Roy Noy, Noelle Marie Coloma Javier

**Affiliations:** Tel Aviv Sourasky Medical Center, Tel Aviv, Israel; Icahn School of Medicine at Mount Sinai, NY, New York, United States

## Abstract

**Background:**

As the global population aged 65 and older continues to grow, so does the number of individuals identifying as LGBTQIA+. Despite this increase LGBTQIA+ health content are often overlooked in medical education, leading to significant disparities in care. Recognizing this gap, the American Geriatrics Society emphasized addressing LGBTQIA+ health disparities as early as 2015.

**Methods:**

Our pilot program, a collaboration between the Brookdale Department of Geriatrics and Palliative Medicine at Mount Sinai in New York and the Tel Aviv Medical Center (TAMC), is designed to address disparities in LGBTQIA+ healthcare. As part of this initiative, a geriatric resident from TAMC completed a four-month observership at Mount Sinai, receiving specialized training tailored to LGBTQIA+ healthcare for older adults. The hands-on curriculum allowed the resident to shadow various services, including geriatric and palliative care, rehabilitation, and specialized clinics such as the Institute for Advanced Medicine and the Center for Transgender Medicine. In addition, the resident engaged in one-on-one educational sessions focused on LGBTQIA+ care.

**Results:**

The outcomes of this pilot program include the creation of a tailored and replicable curriculum for geriatric residency that can be applied to other programs, scholarly peer-reviewed publications, sustainable collaborations, partnerships with advocacy organizations, and the establishment of Tel Aviv’s first LGBTQIA+ geriatric clinic.

**Conclusions:**

Pilot programs like ours hold significant promise for advancing LGBTQIA+ health education and improve the quality of care for older LGBTQIA+ adults, as evidenced by the establishment of Tel Aviv’s first LGBTQIA+ geriatric clinic.

